# Simple Amides of Oleanolic Acid as Effective Penetration Enhancers

**DOI:** 10.1371/journal.pone.0122857

**Published:** 2015-05-26

**Authors:** Barbara Bednarczyk-Cwynar, Danuta Partyka, Lucjusz Zaprutko

**Affiliations:** 1 Department of Organic Chemistry, Faculty of Pharmacy, Poznan University of Medical Sciences, Grunwaldzka Str. 6, 60–780 Poznan, Poland; 2 Department of Technology of Drug Forms, Faculty of Pharmacy, Poznan University of Medical Sciences, Grunwaldzka Str. 6, 60–780 Poznan, Poland; University of East Anglia, UNITED KINGDOM

## Abstract

Transdermal transport is now becoming one of the most convenient and safe pathways for drug delivery. In some cases it is necessary to use skin penetration enhancers in order to allow for the transdermal transport of drugs that are otherwise insufficiently skin-permeable. A series of oleanolic acid amides as potential transdermal penetration enhancers was formed by multistep synthesis and the synthesis of all newly prepared compounds is presented. The synthetized amides of oleanolic acid were tested for their *in vitro* penetration promoter activity. The above activity was evaluated by means of using the Fürst method. The relationships between the chemical structure of the studied compounds and penetration activity are presented.

## Introduction

Administering active substances into the skin constitutes a fundamental pathway of their application which has been known and used for many centuries in order to achieve local action. Because of its simplicity and convenience, this method can also be applied for a substance that can act in the inner parts of the skin or—by being absorbed into the blood—it can cause the systemic effect. However, this pathway is limited because only some substances can be transported through the skin. The substances can reach the deeper skin layers through the intercellular pathway or through the transcellular pathway (by penetration) [1].

In order to improve the rate of transdermal penetration of drugs or cosmetics through the horny layer, physical, chemical and biochemical methods are used [2–8] such as iontophoresis [2], sonophoresis [3], microneedles [4], penetration enhancers [5,6] or liposomal vesicles [7–9]. One of the above mentioned methods involves the addition of certain kinds of substances that can reversibly disrupt horny layer lipid organization, thus making it more permeable. These are known as promoters (accelerators) of transdermal penetration. Penetration enhancers increase skin permeability via various mechanisms, including enhanced solubility, increased partitioning into the stratum corneum, fluidization of its crystalline structure and dissolution of stratum corneum lipids. Besides, deformable colloidal systems enhance migration across the stratum corneum under the influence of hydration or electric forces [6].

The most active enhancers include: hydrocarbons, lower alcohols (such as ethanol), fatty acids (*e*.*g*. oleic acid, lauric acid), fatty alcohols (*e*.*g*. 1-octanol), fatty acid esters (*e*.*g*. isopropyl mirystynate), amines and amides, sulfoxides and their analogues (*e*.*g*. DMSO), pyrrolidones (*e*.*g*. N-methyl-2-pyrrolidone [NMP] and 2-pyrrolidone), tenzides, lipids, laurocapram (Azone) and its derivatives, as well as terpenes and their derivatives [6,10–15].

1-Dodecylazacycloheptan-2-one (Azone) belongs to a large group of transdermal penetration accelerators and is one of the highly effective compound of such activity [6]. It possesses a large polar head group and a lipid alkyl chain, both of which are thought to be necessary for its activity. Its molecule, similarly as NMP, includes an amide nitrogen atom which is also present in some derivatives of oleanolic acid that were synthetized in our laboratory. As is expected from its chemical structure, Azone is a highly lipophilic compound with log P around 6.2. It enhances skin transport of a wide variety of drugs with different lipophilicity [6,10–15].

During last years triterpenes became the subject of scientific research concerning this group of species as active compounds incorporated into different types of vehicles enabling their transdermal transport, but there are only a few works evaluating triterpenes and their derivatives as a transdermal permeation enhancers.

Jaworska et al. [16] studied the formation of oil/water (O/W) nano-emulsions suitable for cosmeceutical application. One of practically non-water soluble triterpenoic acid (name of such compound was not given) was selected as an active compound and incorporated into the stable formulation. The obtained results proved that the nanoemulsion based on caprylic/capric triglycerides with the oil/surfactant ratio O/S = 20:80 and the droplet size r = 25 nm was the most stable one and additionally showed the highest solubilisation capacity for the tested triterpene.

Chen et al. [17] prepared ursolic acid ethosomes and investigated the penetration characteristics of such transdermal vehicles. The ursolic acid ethosomes were prepared by injection method. The triterpene permeation tests in vitro through the rat’s skin were performed in TP-3 diffusion cel. The accumulated permeation amounts of ursolic acid were compared for 10% isopropanolic solution of ursolic acid, as well as for this triterpene liposomes and ethosomes. As the scientists claimed, the encapsulation percentage of the ethosomes was satisfactory and the stability of the ursolic acid ethosomes turned out to be very good. Ethosomes showed to be a good method to enhance the diffusion rate of ursolic acid through the skin of rats.

In 2012 Pino and co-workers [18] tested transdermal permeation of avicins, a family of naturally occurring glycosylated triterpene with a molecular weight more than 2,000 Da through full-thickness porcine skin. As the results shown, avicines, which belong to a family of saponins, exhibit skin permeability comparable to those of small hydrophobic molecules, such as estradiol. Pino and anothers [19] tested also avicines as transdermal permeation enhancers with the appliance of local anesthetics such as hydrochlorides of lidocaine, prilocaine and bupivacaine from aqueous vehicle, across full-thickness porcine skin. Avicins, the triterpenic saponins, in a concentration of 0.5–1%, caused from 2- to 5-fold in comparison to controls sample, without enhancer.

In 1997 the influence of free oleanolic acid on liposomic membranes was described [20]. This triterpene was responsible for causing important changes within the above mentioned membranes and caused their fluidity. Some new oleanolic acid derivatives with lactam and thiolactam systems within the A- or C-ring were also prepared and tested as percutaneous transport promoters *in vitro*. Their activity was comparable with that of N-dodecylcaprolactam (Azone) [21].

## Materials and Methods

### 1. Chemistry

**Fig 1:**
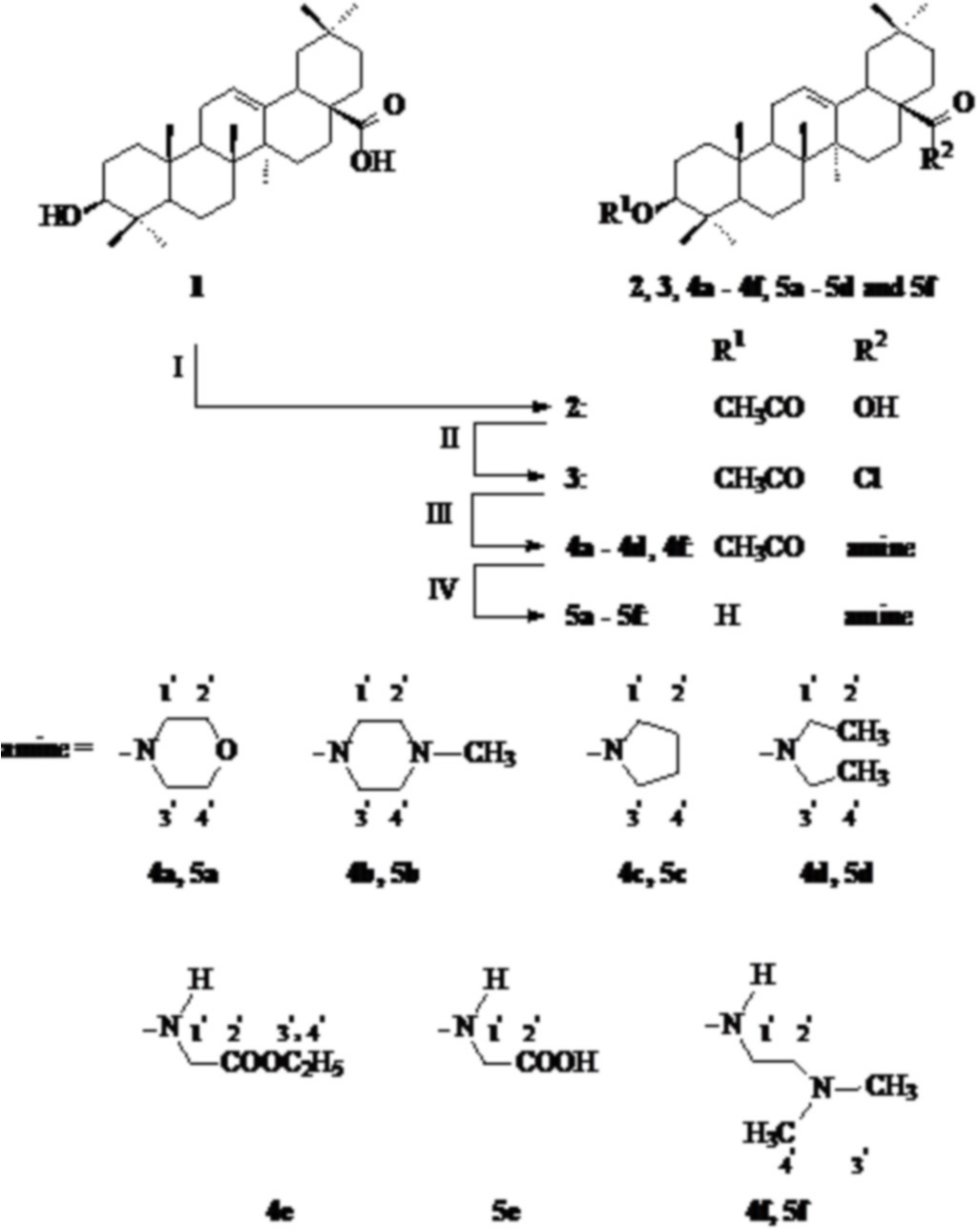
Syntesis of oleanolic acid amides 4a–4f and 5a–5f. Reagents and conditions: I: acetic anhydride, pyridine, r.t.; II: thionyl chloride or oxalyl chloride, r.t.; III: benzene or dichloromethane, amine or glycine ethylester hydrochloride and triethylamine, r.t.; IV: NaOH, ethanol, reflux.

Oleanolic acid, isolated from a natural source (the mistletoe herb, *Viscum album*), was used as the starting material for multistep synthesis of its derivatives. The transformations are presented in the Schemes 1–3. Amide derivatives of oleanolic acid were obtained on the basis of methods known from literature data [22–24]. Oleanolic acid (**1**) was acetylated with a 10-fold excess of acetic anhydride in dried pyridine. The product, with a reversibly blocked hydroxyl group at C-3 position (3-*O*-acetyloleanolic acid, **2**, R^1^ = CH_3_CO, R^2^ = OH), was subjected to thionyl chloride action and the resulting 3-*O*-acetyloleanolic acid chloride in a crude form was transformed into different amides. The reactions of the synthesis of cyclic amides of 3-*O*-acetyloleanolic acid: **4a** – **4c** and aliphatic amides **4d**, **4f** were performed in dried benzene at room temperature (Fig 1) and the amide **4e** was obtained in dichloromethane at room temperature with the use of glycine ethylester hydrochloride and triethylamine as an acceptor of the releasing hydrochloride (Fig 1).

The obtained 3-*O*-acetylamides of oleanolic acid **4a** – **4f** were hydrolized with 5% sodium hydroxide ethanolic solution with short heating (Fig 1). It was proven on the basis of spectral data that in these alkaline conditions the ester bond at C-3 position as well as the ester group of amide **4e** underwent hydrolysis. Alkaline hydrolysis of compound **4e** gave the product **5e**, which was insoluble in biological tests conditions.

**Fig 2:**
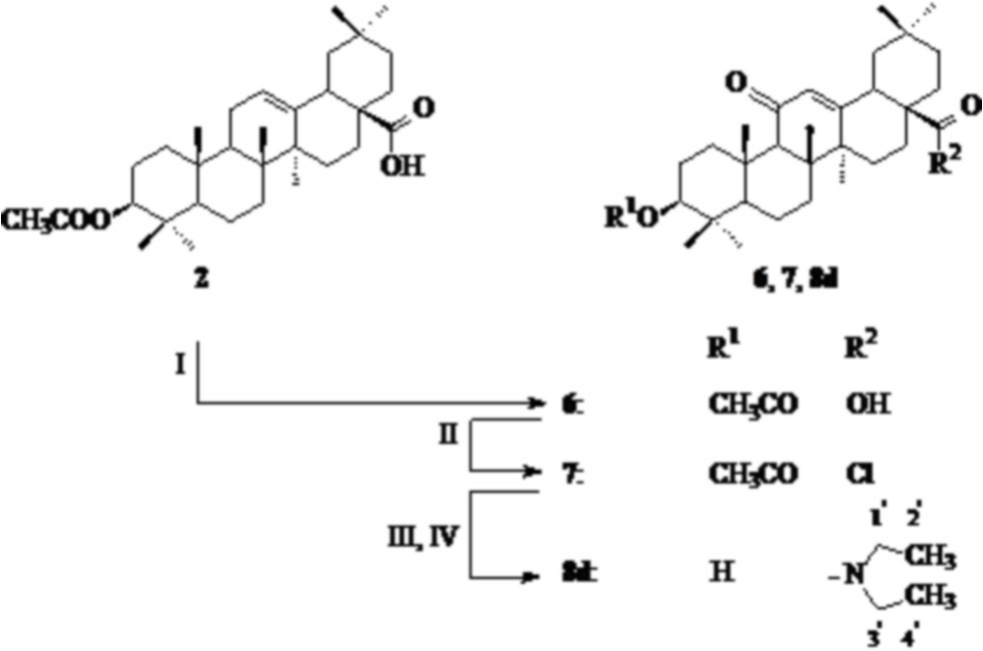
Syntesis of 11-oxooleanolic acid amide 8d. Reagents and conditions: I: sodium dichromate, acetic acid, heating; II: thionyl chloride, r.t.; III: dichloromethane, diethylamine hydrochloride, triethylamine, r.t.; IV: NaOH, ethanol, reflux.

The 11-oxoderivative of oleanolic acid **8d** was obtained by oxidation of acetyloleanolic acid (**2**) with sodium dichromate in acetic acid by applying method known from the literature [25], followed by transformation of the obtained 11-oxoderivative into appriopriate acid chloride (Fig 2) and then into respective amide [22–25].

**Fig 3:**
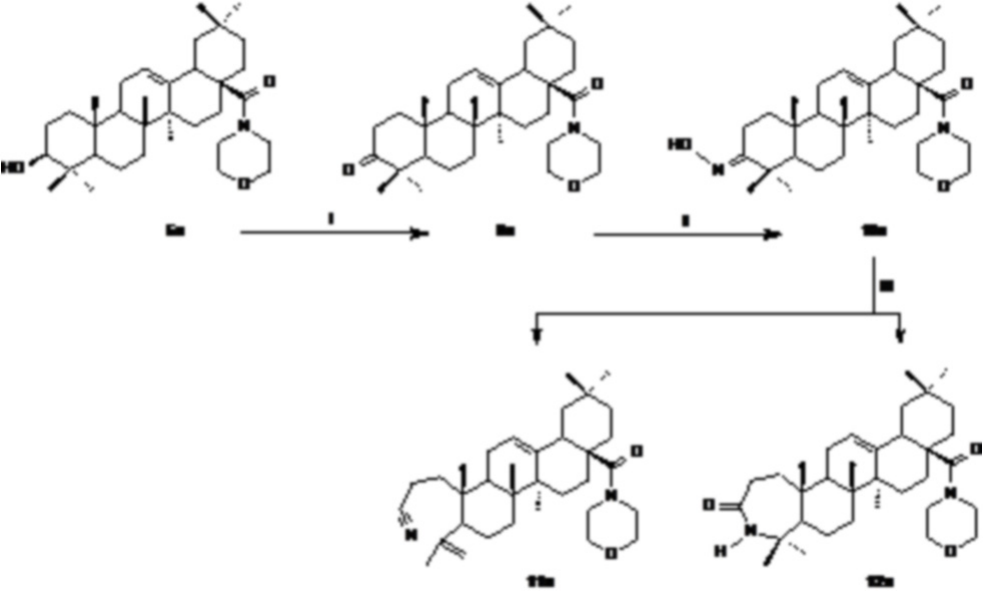
Syntesis of oleanolic acid morpholide derivatives 9a–12a. Reagents and conditions: I: Jones reagent, acetone, r.t.; II: hydroxylamine hydrochloride, sodium acetate, ethanol, reflux; III: phosphoryl chloride, pyridine, r.t.

The derivative of oleanolic acid with the C-3 carbonyl group and the morpholide function (**9a**) was obtained by oxidation of an appropriate amide with free hydroxylic group (**5a**) with Jones reagent (Fig 3) in acetone at room temperature on the basis of the method presented in the literature [26,27].

The morpholides with the nitrile group formed from an opened A ring (**11a**) or with an expanded seven-membered heterocyclic A ring (**12a**) were obtained as is presented in the Scheme 3. The 3-oxoderivative **9a** was first subjected to reaction with hydroxylamine hydrochloride in ethanol [27] and the obtained oxime **10a** was transformed into nitrile **11a** and lactam **12a** under the action of phosphoryl chloride in pyridine by the use of literature method [27].

### 2. In vitro screening of penetration enhancing activity

The aim of the performed test was to evaluate how well newly obtained nitrogen derivatives of oleanolic acid could enhance penetration of progesterone from a hydrogel ointment that was prepared on the basis of Carbopol 980. Progesterone was used as a model of a penetrating active pharmaceutical substance whose pharmacological activity is known and which has a moderate polarity (log P about 4.0). The above pharmaceutical form (hydrogel ointment) contained 1% of progesterone, and 0.5% or 1.0% (if the solubility allowed) of the triterpenic compound (in both cases related to whole formulation) tested as a promoter.

The Fürst method [28] was applied in the *in vitro* tests of progesterone penetration into membrane layers from the hydrogel ointment. This is a simple method allowing to perform quick evaluation of penetration activity of tested compounds. This method consisted of quantitatively determining of progesterone absorbed into the structure which was constructed of five lipophilic membranes. The ointment was spread on the surface of the first membrane (4 cm^2^) of the above mentioned system and left in a thermostat at 37°C for 1h. After this period of time the content of the therapeutic substance was determined separately for each of the five lipophilic membranes. The spectrophotometric method using the colour reaction of progesterone and isonicotinic acid complex formation was applied in this order. The penetration tests were repeated 6 times.

The artificial lipophilic membranes were prepared in our laboratory and they were made from dodecanol as a lipid phase and collodium as a colloxylinic matrix supporting the membranes. The thickness of all of the obtained membranes was determined on the basis of the Richter method [29] in which the spectrum of the interference wave was recorded for each membrane in the infrared range.

## Results and Discussion

In this work a series of oleanolic acid derivatives with the amide function was prepared as potential transdermal penetration enhancers. These compounds’ ability to enhance the penetration of progesterone as a model substance through the system of five lipophilic membranes was tested. All of the prepared oleanolic acid amide derivatives positively influenced the amount of the model substance (progesterone) that was absorbed in five synthetic membranes. A markedly increased amount of absorbed progesterone in all of the membranes, higher than for Azone, was observed for amides: **4b**, **5b** (both with the N-methylpiperazine moiety and the acetoxy or the hydroxy group at C-3 position), **4a**, **5a** (both with the morpholine ring), **4d** (with the N,N-diethylamide function) and **4f** (having two nitrogen atoms within the amide function and the acetoxy group at C-3 position). Because the amides with the morpholine ring (**4a** and **5a**) seemed to be particularly active compounds, further modifications of the C-3 hydroxyl function were performed and ketone (**9a**), oxime (**10a**), nitrile (**11a**) and lactam (**12a**) were obtained. Among the above compounds, lactam **12a** exhibited the best results. In most of the described cases the increase of the promoter concentration in the ointment from 0.5% to 1.0% caused an increase in the total amount of progesterone. In general, replacement of the C-3 acetyl group with a free hydroxyl function caused an insignificant decrease in the total amount of progesterone in the set of lipophilic membranes. Only the hydrolysis of the acetyl function of compound **4c** leading to compound **5c** caused an increase in the total amount of absorbed progesterone in the five membranes. The introduction of an additional carbonyl group at C-11 position (compound **8d**) did not cause a significant increase in the total amount of progesterone in the set of membranes. Oxidation of the C-3 hydroxyl group of compound **5a**, leading to ketone **9a** also had no important influence on the total amount of model substance absorbed into the five membranes. The transformation of the 3-oxo function of derivative **9a** to the 3-hydroxyimino group (within triterpene **10a**) did not increase the amount of absorbed progesterone. The rearrangement of oxime (**10a**) into lactam (**12a**) led to an improvement in promoting activity of triterpene.

In order to compare the influence of triterpenes on the depth of progesterone penetration, the amount of the absorbed model drug was determined for membranes 1–3 (meaning the “external layer” of the epidermis model), 4–5 (the „internal layer” of the epidermis model) and 1–5 (the „whole” epidermis model).

The presence of Azone as a transdermal promoter played a significant role in the increase of the total amount of absorbed progesterone and the amount of progesterone that permeated into the most external membranes (1–3), but at the same time it caused an important decrease in the amount of progesterone in the internal membranes (4–5). Almost all of the obtained amides of oleanolic acid caused an increase in the amount of the absorbed model substance in the “external layer” of the epidermis model as well as in this model’s “internal layer”. Amide **4a** in a concentration of 1.0% caused the highest increase in the amount of absorbed progesterone in membranes 1–3: the relative increase in the amount of the absorbed drug was over 40% in comparison to about 32% for Azone. At the same time, two triterpenes in a concentration of 0.5% (**4b** and **5b**) and four derivatives in a concentration of 1.0% (**9a**, **12a**, **5c** and **4d**) caused a more than 50% increase in the amount of the absorbed progesterone into more deeply located membranes. Replacement of the C-3 hydroxyl group with the carbonyl one or with the hydroxyimino function led to an important improvement in promoting activity of triterpene within the more internal membranes. A further increase in the amount of progesterone in the deeper membranes was caused by the transformation of oxime **10a** (about 40%) into lactam **12a** (almost 66%).

## Experimental

### 1. Chemistry

#### 1.1. General

All solvents and reagents were pure for analysis grade and purchased from Sigma-Aldrich. For reactions in anhydrous conditions the solvents were purified and dried by using general methods described in literature [30]. The thin layer chromatography (TLC) analysis (reactions progress and the level of compounds purity) was conducted on HPTLC aluminum sheets covered with silicagel 60 F245 and benzene with ethyl acetate in a volume ratio of 4: 1 as the eluent. The chromatograms were visualized by being sprayed with 10% ethanolic solution of sulfuric acid and heating the plates at about 110°C for a few minutes. The column chromatography was performed using silicagel 60 (0.063–0.200 mm, 70–230 mesh).

Melting points were determined in open capillaries using the Büchi apparatus.

IR spectra were recorded using the Specord IR—75 spectrophotometer, for 0.5% mixtures of the tested compounds and KBr. NMR spectra for hydrogen atoms (^1^H) and for carbon atoms (^13^C) were recorded in deuterated chloroform or DMSO solutions on the Varian Gemini 300 VT apparatus, for frequencies 300 MHz and 75 MHz, respectively, with TMS as an internal standard. The multiplicities of signals in ^1^H NMR spectra were marked as follows: s—singlet, br/s—broad singlet, t—triplet, dd—doublet of doublets, m—multiplet. Mass spectra (MS) and high resolution mass spectra (HR MS) were recorded using the AMD 402 spectrometer with electroionization. The elucidation of the chemical structures was based on IR, ^1^H NMR, ^13^C NMR, MS and HR MS analysis.

#### 1.2. General method for synthesis of amides 4a – 4d and 4f

Amine (0.01 mol) was added dropwise to a vigorously stirred solution of crude 3β-acetoxyolean-12-en-28-oic acid chloride **3** (obtained from 500 mg, 0.001 mol of 3β-acetoxyolean-12-en-28-oic acid **2**) in dried benzene (10 mL) and stirred up to the total consumption of the substrate (TLC control). The resulting solid was filtered off, the filtrate was washed with water, dried and evaporated. The obtained residue was crystallized from ethanol to afford the title amides **4a** – **4d** and **4f**.

**3β-Acetoxyolean-12-en-28-oic acid morpholide 4a**: C_36_H_57_NO_4_; mol. mass: 567.85; yield: 549 mg (96.6%); m.p.: 238–239°C; **IR** (KBr, ν, cm^-1^): 1720 (C = O, CH_3_COO), 1630 (C = O, amide); ^**1**^ **H NMR** (CHCl_3_, δ, ppm): 5.27 (1H, t, *J* = 3.4 Hz, C_12_-H), 4.49 (1H, dd, *J* = 7.0 and 8.9 Hz, C_3_-H_α_), 3.70–3.57 (8H, m, morpholine ring), 2.05 (3H, s, CH_3_COO), 1.14, 0.93, 0.92, 0.90, 0.86, 0.85 and 0.74 (21 H, 7 x s, 7 x CH_3_); ^**13**^ **C NMR** (CHCl_3_, δ, ppm): 175.1 (C_q_, C-28), 171.0 (C_q_, CH_3_COO), 144.6 (C_q_, C-13), 121.5 (CH, C-12), 80.9 (CH, C-3), 2 x 66.9, 46.3 and 46.0 (4 x CH_2_, morpholine ring), 47.4 (C_q_, C-17), 21.3 (CH_3_, CH_3_COO); **DEPT**: 8 x CH_3_, 14 x CH_2_, 5 x CH (36 x C atoms); **MS** (m/z, %): 567.4 (68.8, M^+•^); **HR MS**: calcd. for C_36_H_57_NO_4_: 567.4287, found: 567.4285.

**3β-Acetoxyolean-12-en-28-oic acid N-methylpiperazide 4b**: C_37_H_60_N_2_O_3_; mol. mass: 580.90; yield: 541 mg (93.2%); m.p.: 218–220°C; **IR** (KBr, ν, cm^-1^): 1710 (C = O, CH_3_COO), 1600 (C = O, amide); ^**1**^ **H NMR** (CHCl_3_, δ, ppm): 5.26 (1H, t, *J* = 3.5 Hz, C_12_-H), 4.49 (1H, dd, *J* = 7.0 and 8.8 Hz, C_3_-H_α_), 3.70–3.59 and 2.41–2.31 (2 x 4H, 2 x m, piperazine ring), 2.29 (3H, s, >N–CH_3_), 2.05 (3H, s, CH_3_COO), 1.13, 0.93, 0.92, 0.90, 0.86, 0.85 and 0.74 (21 H, 7 x s, 7 x CH_3_); ^**13**^ **C NMR** (CHCl_3_, δ, ppm): 174.9 (C_q_, C-28), 170.9 (C_q_, CH_3_COO), 144.8 (C_q_, C-13), 121.3 (CH, C-12), 80.9 (CH, C-3), 55.2 x 2 and 38.0 x 2 (4 x CH_2_, piperazine ring), 47.6 (C_q_, C-17), 46.0 (CH_3_, >N–CH_3_), 21.3 (CH_3_, CH_3_COO); **DEPT**: 9 x CH_3_, 14 x CH_2_, 5 x CH (37 x C atoms); **MS** (m/z, %): 580.7 (3.6, M^+•^); **HR MS**: calcd. for C_37_H_60_N_2_O_3_: 580.4604, found: 580.4600.

**3β-Acetoxyolean-12-en-28-oic acid pyrrolidide 4c**: C_36_H_57_NO_3_; mol. mass: 551.85; yield: 507 mg (91.9%); m.p.: 212–213°C; **IR** (ν, cm^-1^): 1720 (C = O, CH_3_COO), 1710 (C = O, amide); ^**1**^ **H NMR** (CHCl_3_, δ, ppm): 5.26 (1H, t, *J* = 3.5 Hz, C_12_-H), 4.49 (1H, dd, *J* = 7.0 and 8.9 Hz, C_3_-H_α_), 3.55–3.44 and 1.73–1.54 (2 x 4H, 2 x m, pyrrolidine ring), 2.04 (3H, s, CH_3_COO), 1.14, 0.94, 0.92, 0.90, 0.86, 0.85 and 0.72 (21 H, 7 x s, 7 x CH_3_); ^**13**^ **C NMR** (CHCl_3_, δ, ppm): 175.1 (C_q_, C-28), 170.9 (C_q_, CH_3_COO), 144.8 (C_q_, C-13), 121.6 (CH, C-12), 80.9 (CH, C-3), 47.3 (C_q_, C-17), 46.1 x 2 and 27.5 x 2 (4 x CH_2_, pyrrolidine ring), 21.2 (CH_3_, CH_3_COO); **DEPT**: 8 x CH_3_, 14 x CH_2_, 5 x CH (36 x C atoms); **MS** (m/z, %): 551.6 (99.9, M^+•^); **HR MS**: calcd. for C_36_H_57_NO_3_: 551.4338, found: 551.4340.

**3β-Acetoxyolean-12-en-28-oic acid N,N-diethylamide 4d**: C_36_H_59_NO_3_; mol. mass: 553.87; yield: 512 mg (92.5%); m.p.: 224–226°C; **IR** (KBr, ν, cm^-1^): 1720 (C = O, CH_3_COO), 1705 (C = O, amide); ^**1**^ **H NMR** (CHCl_3_, δ, ppm): 5.26 (1H, t, *J* = 3.6 Hz, C_12_-H), 4.49 (1H, dd, *J* = 7.0 and 8.9 Hz, C_3_-H_α_), 3.49–3.26 and 1.99–1.81 (2 x 2H, 2 x m,—N< (CH_2_–CH_3_)_2_), 2.04 (3H, s, CH_3_COO), 1.14 (6H, s,—N<(CH_2_–CH_3_)_2_), 1.12, 0.93, 0.92, 0.89, 0.86, 0.85 and 0.78 (21 H, 7 x s, 7 x CH_3_); ^**13**^ **C NMR** (CHCl_3_, δ, ppm): 174.9 (C_q_, C-28), 170.9 (C_q_, CH_3_COO), 145.0 (C_q_, C-13), 121.1 (CH, C-12), 80.9 (CH, C-3), 47.7 (C_q_, C-17), 46.7 x 2 (CH_2_ x 2,—N<(CH_2_–CH_3_)_2_), 21.3 (CH_3_, CH_3_COO), 16.6 x 2 (CH_3_ x 2,—N<(CH_2_–CH_3_)_2_); **DEPT**: 10 x CH_3_, 12 x CH_2_, 5 x CH (36 x C atoms); **MS** (m/z, %): 553.7 (34.7, M^+•^); **HR MS**: calcd. for C_36_H_59_NO_3_: 553.4492, found: 553.4490.

**3β-Acetoxyolean-12-en-28-oic acid N’,N’-dimethylaminoethylamide 4f**: C_36_H_60_N_2_O_3_; mol. mass: 568.88; yield: 535 mg (94.1%); m.p.: 163–165°C; **IR** (KBr, ν, cm^-1^): 3400 (N–H), 1720 (C = O, CH_3_COO), 1630 (C = O, amide); ^**1**^ **H NMR** (DMSO, δ, ppm): 7.71 (1H, t, *J* = 5.2 Hz, N–H), 5.23 (1H, s, C_12_-H), 4.49 (1H, dd, *J* = 4.6 and 11.1 Hz, C_3_-H_α_), 2.72 (6H, s,—NH–CH_2_–CH_2_–N<(CH_3_ )_2_), 2.02 (3H, s, CH_3_COO), 1.62–1.44 (4H, m,—NH–CH_2_ –CH_2_–N<(CH_3_)_2_), 1.11, 0.89, 0.88, 0.87, 0.81, 0.80, 0.67 (21 H, 7 x s, 7 x CH_3_); ^**13**^ **C NMR** (DMSO, δ, ppm): 177.1 (C_q_, C-28), 170.9 (C_q_, CH_3_COO), 143.9 (C_q_, C-13), 121.5 (CH, C-12), 79.9 (CH, C-3), 56.0 x 2 (2 x CH_2_,—NH–CH_2_ –CH_2_–N<(CH_3_)_2_), 45.4 (C_q_, C-17), 42.5 x 2 (2 x CH_3_,—NH–CH_2_–CH_2_–N<(CH_3_ )_2_), 21.0 (CH_3_, CH_3_COO); **DEPT**: 10 x CH_3_, 12 x CH_2_, 5 x CH (36 x C atoms); **MS** (m/z, %): 568.4 (6.01, M^+•^); **HR MS**: calcd. for C_36_H_60_N_2_O_3_: 568.4603, found: 568.4606.

#### 1.3. Synthesis of amide 4e

A solution of glycine ethylester hydrochloride (0.004 mol) and triethylamine (0.008 mol) in dichloromethane (10 mL) was added to a vigorously stirred solution of crude 3β-acetoxyolean-12-en-28-oic acid chloride **3** (obtained from 500 mg, 0.001 mol of 3β-acetoxyolean-12-en-28-oic acid **2**) in dried dichloromethane (10 mL) and left at room temperature overnight (TLC control). The resulting mixture was washed with water, dried and subjected to column chromatography with silicagel (benzene and ethyl acetate, volume ratio of 4:1). After evaporation the obtained residue was crystallized from ethanol to afford the title amide **4e**.

**3β-Acetoxyolean-12-en-28-oic acid ethoxycarbonylmethylamide 4e**: C_36_H_57_NO_5_; mol. mass: 583.85; yield: 424 mg (72.7%); m.p.: 169–170°C (ethanol); **IR** (KBr, ν, cm^-1^): 3390 (N–H), 1750 (C = O, COOC_2_H_5_), 1720 (C = O, CH_3_COO), 1630 (C = O, amide); ^**1**^ **H NMR** (CHCl_3_, δ, ppm): 6.57 (1H, dd, *J* = 3.7 and 5.1 Hz, N–H), 5.46 (1H, t, *J* = 3.3 Hz, C_12_-H), 4.49 (1H, dd, *J* = 7.9 and 7.9 Hz, C_3_-H_α_), 4.22 (2H, dd, *J* = 7.1 and 14.3 Hz,—NH–CH_2_–COO–CH_2_–CH_3_), 3.74 (2H, dd, *J* = 5.2 and 15.5 Hz,—NH–CH_2_–COO–CH_2_–CH_3_), 2.05 (3H, s, CH_3_COO), 1.29 (3H, s,—NH–CH_2_–COO–CH_2_–CH_3_), 1.24, 1.16, 0.92, 0.92, 0.86, 0.85, 0.70 (21 H, 7 x s, 7 x CH_3_); ^**13**^ **C NMR** (CHCl_3_, δ, ppm): 178.2 (C_q_, C-28), 171.0 and 170.0 (2 x C_q_, CH_3_COO and—NH–CH_2_–COO–CH_2_–CH_3_), 144.2 (C_q_, C-13), 123.1 (CH, C-12), 80.8 (CH, C-3), 61.4 (CH_2_,—NH–CH_2_–COO–CH_2_–CH_3_), 46.5 (CH_2_,—NH–CH_2_–COO–CH_2_–CH_3_), 46.2 (C_q_, C-17), 21.2 (CH_3_, CH_3_COO), 14.1 (CH_3_,—NH–CH_2_–COO–CH_2_–CH_3_); **DEPT**: 9 x CH_3_, 12 x CH_2_, 5 x CH (36 x C atoms); **MS** (m/z, %): 583.5 (11.7, M^+•^); **HR MS**: calcd. for C_36_H_57_NO_5_: 583.4236, found: 583.4230.

#### 1.4. General metod for synthesis of amides 5a – 5f

A saturated solution of 3β-acetoxyolean-12-en-28-oic acid amide **4a** – **4f** (0.001 mol) in 5% ethanolic solution of NaOH was heated under reflux until the total consumption of the substrate (TLC control, about 30 min). The resulting mixture was cooled and poured into a 5-fold volume of water slightly acidified with HCl. The obtained precipitate was filtered off, washed with water and dried. The received residue was crystallized from ethanol or precipitated with water from ethanolic solution to afford the title amides **5a** – **5f**.

**3β-Hydroxyolean-12-en-28-oic acid morpholide 5a**: C_34_H_55_NO_3_; mol. mass: 525.82; yield: 502 mg (95.5%); m.p.: 230–231°C (ethanol with water); **IR** (KBr, ν, cm^-1^): 3445 (O–H), 1625 (C = O); ^**1**^ **H NMR** (CHCl_3_, δ, ppm): 5.27 (1H, t, *J* = 3.4 Hz, C_12_-H), 3.70–3.58 (8H, m, morpholine ring), 3.22 (1H, dd, *J* = 3.8 and 7.7 Hz, C_3_-H_α_), 1.14, 0.99, 0.93, 0.90, 0.89, 0.78, 0.73 (21 H, 7 x s, 7 x CH_3_); ^**13**^ **C NMR** (CHCl_3_, δ, ppm): 175.0 (C_q_, C-28), 144.5 (C_q_, C-13), 121.6 (CH, C-12), 79.0 (CH, C-3), 2 x 67.0, 46.5 and 46.2 (4 x CH_2_, morpholine ring), 47.9 (C_q_, C-17); **DEPT**: 7 x CH_3_, 14 x CH_2_, 5 x CH (34 x C atoms); **MS** (m/z, %): 525.4 (63.7, M^+•^); **HR MS**: calcd. for C_34_H_55_NO_3_: 525.4182, found: 525.4189.

**3β-Hydroxyolean-12-en-28-oic acid N-methylpiperazide 5b**: C_35_H_58_N_2_O_2_; mol. mass: 538.86; yield: 512 mg (95.1%); m.p.: 244–245°C; **IR** (KBr, ν, cm^-1^): 3450 (O–H), 1600 (C = O); ^**1**^ **H NMR** (CHCl_3_, δ, ppm): 5.26 (1H, t, *J* = 3.5 Hz, C_12_-H), 3.69–3.60 and 2.40–2.30 (2 x 4H, 2 x m, piperazine ring), 3.21 (1H, dd, *J* = 3.8 and 7.7 Hz, C_3_-H_α_), 2.29 (3H, s, >N–CH_3_), 1.13, 0.93, 0.92, 0.90, 0.86, 0.85, 0.74 (21 H, 7 x s, 7 x CH_3_); ^**13**^ **C NMR** (CHCl_3_, δ, ppm): 174.8 (C_q_, C-28), 144.9 (C_q_, C-13), 121.3 (CH, C-12), 79.0 (CH, C-3), 55.2 x 2 and 38.0 x 2 (4 x CH_2_, piperazine ring), 47.5 (C_q_, C-17), 46.0 (CH_3_, >N–CH_3_); **DEPT**: 8 x CH_3_, 14 x CH_2_, 5 x CH (35 x C atoms); **MS** (m/z, %): 538.5 (3.9, M^+•^); **HR MS**: calcd. for C_35_H_58_N_2_O_2_: 538.4498, found: 538.4501.

**3β-Hydroxyolean-12-en-28-oic acid pyrrolidide 5c**: C_34_H_55_NO_2_; mol. mass: 509.82; yield: 484 mg (94.9%); m.p.: 227–229°C; **IR** (KBr, ν, cm^-1^): 3480 (O–H), 1695 (C = O); ^**1**^ **H NMR** (CHCl_3_, δ, ppm): 5.27 (1H, t, *J* = 3.5 Hz, C_12_-H), 3.55–3.44 and 1.73–1.54 (2 x 4H, 2 x m, pyrrolidine ring), 3.20 (1H, dd, *J* = 3.8 and 7.8 Hz, C_3_-H_α_), 1.16, 0.94, 0.90, 0.89, 0.86, 0.85, 0.70 (21 H, 7 x s, 7 x CH_3_); ^**13**^ **C NMR** (CHCl_3_, δ, ppm): 175.3 (C_q_, C-28), 144.4 (C_q_, C-13), 121.8 (CH, C-12), 78.9 (CH, C-3), 47.3 (C_q_, C-17), 46.0 x 2 and 27.4 x 2 (4 x CH_2_, pyrrolidine ring), **DEPT**: 7 x CH_3_, 14 x CH_2_, 5 x CH (34 x C atoms); **MS** (m/z, %): 509.6 (95.9, M^+•^); **HR MS**: calcd. for C_34_H_55_NO_2_: 509.4232, found: 509.4237.

**3β-Hydroxyolean-12-en-28-oic acid N,N-diethylamide 5d**: C_34_H_57_NO_2_; mol. mass: 511.83; yield: 481 mg (93.9%); m.p.: 198–199°C; **IR** (KBr, ν, cm^-1^): 3480 (O–H), 1700 (C = O); ^**1**^ **H NMR** (CHCl_3_, δ, ppm): 5.26 (1H, t, *J* = 3.5 Hz, C_12_-H), 3.50–3.26 and 1.99–1.82 (2 x 2H, 2 x m,—N<(CH_2_–CH_3_)_2_), 3.20 (1H, dd, *J* = 3.8 and 7.8 Hz, C_3_-H_α_), 1.14 (6H, s,—N<(CH_2_–CH_3_)_2_), 1.12, 0.92, 0.91, 0.90, 0.86, 0.84, 0.78 (21 H, 7 x s, 7 x CH_3_); ^**13**^ **C NMR** (CHCl_3_, δ, ppm): 175.0 (C_q_, C-28), 144.9 (C_q_, C-13), 121.3 (CH, C-12), 78.9 (CH, C-3), 47.7 (C_q_, C-17), 46.6 x 2 (CH_2_ x 2,—N<(CH_2_–CH_3_)_2_), 16.7 x 2 (CH_3_ x 2,—N<(CH_2_–CH_3_)_2_); **DEPT**: 9 x CH_3_, 12 x CH_2_, 5 x CH (34 x C atoms); **MS** (m/z, %): 511.7 (35.2, M^+•^); **HR MS**: calcd. for C_34_H_57_NO_2_: 511.4389, found: 511.4392.

**3β-Hydroxyolean-12-en-28-oic acid hydroxycarbonylmethylamide 5e**: C_32_H_51_NO_4_; mol. mass: 513.76; yield: 487 mg (94.9%); m.p.: 238–241°C (precipit. with water from ethanolic sol.); **IR** (KBr, ν, cm^-1^): 3490 (O–H), 3400 (N–H), 1710 (C = O, COOH), 1635 (C = O, amide); ^**1**^ **H NMR** (DMSO, δ, ppm): 12.37 (1H, br/s, COOH), 7.58 (1H, t, *J* = 5.4 Hz, N–H), 5.19 (1H, s, C_12_-H), 3.74 (1H, dd, *J* = 5.9 and 17.3 Hz) and 3.54 (1H, dd, *J* = 5.2 and 17.2 Hz,—NH–CH_2_–COOH), 2.99 (1H, dd, *J* = 6.3 and 9.0 Hz, C_3_-H_α_), 1.08, 0.89, 0.88, 0.88, 0.84, 0.67, 0.64 (21 H, 7 x s, 7 x CH_3_); ^**13**^ **C NMR** (DMSO, δ, ppm): 176.7 (C_q_, C-28), 171.4 (C_q_,—NH–CH_2_–COOH), 143.9 (C_q_, C-13), 121.5 (CH, C-12), 76.8 (CH, C-3), 46.0 (CH_2_,—NH–CH_2_–COOH), 45.2 (C_q_, C-17); **DEPT**: 7 x CH_3_, 11 x CH_2_, 5 x CH (32 x C atoms); **MS** (m/z, %): 513.5 (20.7, M^+•^); **HR MS**: calcd. for C_32_H_51_NO_4_: 513.3818, found: 513.3812.

**3β-Hydroxyolean-12-en-28-oic acid N’,N’–dimethylaminoethylamide 5f**: C_34_H_58_N_2_O_2_; mol mass: 526.85; yield: 486 mg (92.2%); m.p.: 122–124°C; **IR** (KBr, ν, cm^-1^): 3485 (O–H), 3400 (N–H), 1630 (C = O); ^**1**^ **H NMR** (DMSO, δ, ppm): 7.75 (1H, t, *J* = 5.3 Hz, N–H), 5.24 (1H, s, C_12_-H), 3.23 (1H, d, *J* = 4.6 Hz, C_3_-H_α_), 2.74 (6H, s,—NH–CH_2_–CH_2_–N<(CH_3_ )_2_), 1.67–1.46 (4H, m,—NH–CH_2_ –CH_2_–N<(CH_3_)_2_), 1.09, 0.89, 0.88, 0.87, 0.85, 0.68, 0.67 (21 H, 7 x s, 7 x CH_3_); ^**13**^ **C NMR** (DMSO, δ, ppm): 177.4 (C_q_, C-28), 143.9 (C_q_, C-13), 121.7 (CH, C-12), 78.9 (CH, C-3), 56.1 x 2 (2 x CH_2_,—NH–CH_2_ –CH_2_–N<(CH_3_)_2_), 45.4 (C_q_, C-17), 42.7 x 2 (2 x CH_3_,—NH–CH_2_–CH_2_–N<(CH_3_ )_2_); **DEPT**: 9 x CH_3_, 12 x CH_2_, 5 x CH (34 x C atoms); **MS** (m/z, %): 526.4 (19.5, M^+•^); **HR MS**: calcd. for C_34_H_58_N_2_O_2_: 526.4498, found: 526.4491.

#### 1.5. Synthesis of amide 8d

Synthesis was performed from 3β-acetoxy-11-oxoolean-12-en-28-oic acid chloride (**7**) as described in 1.2. The crude product was subjected to column chromatography with silicagel (benzene and ethyl acetate, volume ratio of 9:1). After evaporation the obtained residue was crystallized to afford the title amide **8d**.

**3β-Acetoxy-11-oxoolean-12-en-28-oic acid N,N-diethylamide 8d**: C_36_H_57_NO_4_; mol mass: 567.85; yield: 378 mg (66.6%); m.p.: 270–272 ^o^C (decomp.); **IR** (KBr, ν, cm^-1^): 1720 (C = O, CH_3_COO); 1710 (C = O, amide), 1650 (C = O at C-11); ^**1**^ **H NMR** (CHCl_3_, δ, ppm): 5.66 (1H, s, C_12_-H), 4.50 (1H, dd, *J* = 7.0 and 8.8 Hz, C_3_-H_α_), 3.50–3.26 and 2.01–1.81 (2 x 2H, 2 x m,—N<(CH_2_–CH_3_)_2_), 2.05 (3H, s, CH_3_COO), 1.14 (6H, s,—N<(CH_2_–CH_3_)_2_), 1.11, 0.92, 0.92, 0.90, 0.86, 0.82, 0.75 (21 H, 7 x s, 7 x CH_3_); ^**13**^ **C NMR** (CHCl_3_, δ, ppm): 199.8 (C_q_, C-11); 175.0 (C_q_, C-28), 170.7 (C_q_, CH_3_COO), 168.3 (C_q_, C-13); 127.9 (CH, C-12), 81.0 (CH, C-3), 47.6 (C_q_, C-17), 46.6 x 2 (CH_2_ x 2,—N<(CH_2_–CH_3_)_2_), 21.2 (CH_3_, CH_3_COO), 16.5 x 2 (CH_3_ x 2,—N<(CH_2_–CH_3_)_2_); **DEPT**: 10 x CH_3_, 11 x CH_2_, 5 x CH (36 x C atoms); **MS** (m/z, %): 567.6 (32.1, M^+•^); **HR MS**: calcd. for C_36_H_57_NO_4_: 567.4287, found: 567.4292.

#### 1.6. Synthesis of amide 9a

Jones’ reagent was added dropwise with small excess to a vigorously stirred solution of 3β-hydroxyolean-12-en-28-oic acid morpholide (**5a**, 0.001 mol) in acetone at room temperature. The resulting mixture was stirred up to the total consumption of the substrate (TLC control, about 30 min), then isopropyl alcohol was added dropwise and the obtained suspension was filtered off; the filtrate was poured into a 5-fold volume of water slightly acidified with HCl. The formed precipitate was filtered off, washed with water and dried. The obtained residue was crystallized from ethanol to afford the title amide **9a**.

**3-Oxoolean-12-en-28-oic acid morpholide 9a**: C_34_H_53_NO_3_; mol mass: 523.80; yield: 495 mg (94.5%); m.p.: 215–218°C; **IR** (KBr, ν, cm^-1^): 1690 (C = O, C-3), 1625 (C = O, amide); ^**1**^ **H NMR** (CHCl_3_, δ, ppm): 5.29 (1H, t, *J* = 3.4 Hz, C_12_-H), 3.71–3.57 (8H, m, morpholine ring), 1.15, 1.09, 1.04, 1.03, 0.93, 0.90, 0.79 (21 H, 7 x s, 7 x CH_3_); ^**13**^ **C NMR** (CHCl_3_, δ, ppm): 217.0 (C_q_, C-3), 175.0 (C_q_, C-28), 144.6 (C_q_, C-13), 121.4 (CH, C-12), 66.9 x 2, 46.4 and 46.2 (4 x CH_2_, morpholine ring), 47.5 (C_q_, C-17); **DEPT**: 7 x CH_3_, 14 x CH_2_, 4 x CH (34 x C atoms); **MS** (m/z, %): 523.5 (100.0, M^+•^); **HR MS**: calcd. for C_34_H_53_NO_3_: 523.4025, found: 523.4022.

#### 1.7. Synthesis of amide 10a

Hydroxylamine hydrochloride (350 mg, 0.005 mol) and sodium acetate (660 mg, 0.008 mol) were added to a saturated, hot solution of 3-oxoolean-12-en-28-oic acid morpholide (**9a**, 0.001 mol) in ethanol and the resulting mixture was refluxed up to total consumption of the substrate (TLC control, about 30 min), cooled and poured into a 5-fold volume of water slightly acidified with HCl. The formed precipitate was filtered off, washed with water and dried. The obtained residue was crystallized from ethanol to afford the title amide **10a**.

**3-Hydroxyiminoolean-12-en-28-oic acid morpholide 10a**: C_34_H_54_N_2_O_3_; mol mass: 538.81; yield: 467 mg (86.7%); m.p.: 210–211°C; **IR** (KBr, ν, cm^-1^): 3445 (O–H), 1625 (C = O); ^**1**^ **H NMR** (CHCl_3_, δ, ppm): 9.03 (1H, s, N-OH); 5.28 (1H, t, *J* = 3.1 Hz, C_12_-H), 3.71–3.57 (8H, m, morpholine ring), 1.16, 1.12, 1.05, 1.03, 0.93, 0.90, 0.77 (21 H, 7 x s, 7 x CH_3_); ^**13**^ **C NMR** (CHCl_3_, δ, ppm): 175.2 (C_q_, C-28), 167.0 (C_q_, C-3), 144.7 (C_q_, C-13), 121.5 (CH, C-12), 66.9 x 2, 46.3 and 46.0 (4 x CH_2_, morpholine ring), 47.4 (C_q_, C-17); **DEPT**: 7 x CH_3_, 14 x CH_2_, 4 x CH (34 x C atoms); **MS** (m/z, %): 538.6 (100.0, M^+•^); **HR MS**: calcd. for C_34_H_54_N_2_O_3_: 538.4134, found: 538.4132.

#### 1.8. Synthesis of amides 11a and 12a

Phosphoryl chloride (POCl_3_, 0.28 mL, 460 mg, 0.003 mol) was added dropwise with stirring to a saturated solution of 3-hydroxyiminoolean-12-en-28-oic acid morpholide (**10a**, 539 mg, 0.001 mol) in anhydrous pyridine. The resulting mixture was left in room temperature overnight, poured into a 5-fold volume of water slightly acidified with HCl. The precipitate was filtered off, washed with water and dried. The obtained residue was subjected to column chromatography with silicagel (benzene and acetone, volume ratio of 1:1, next acetone). After evaporation the obtained residues were crystallized to afford the title amides: **11a** and **12a**.

**3-Nitrile-3,4-sekoolean-4(23),12-dien-28-oic acid morpholide 11a** (afforded from benzene and acetone fraction): C_34_H_52_N_2_O_2_; mol mass: 520.80; yield: 107 mg (20.5%); m.p.: 195–197°C (*n*-hexane); **IR** (KBr, ν, cm^-1^): 3060 (C = C), 2240 (C≡N), 1630 (C = O); ^**1**^ **H NMR** (CHCl_3_, δ, ppm): 5.28 (1H, t, *J* = 3.1 Hz, C_12_-H), 4.90 (1H, s, C_23_-H), 4.66 (1H, s, C_23_-H), 3.72–3.52 (4 x 2H, m, morpholine ring), 1.74 (3H, s, C_24_-H), 1.15, 0.97, 0.92, 0.90, 0.80 (15 H, 5 x s, 5 x CH_3_); ^**13**^ **C NMR** (CHCl_3_, δ, ppm): 175.1 (C_q_, C-28), 146.8 (C_q_, C-4), 144.9 (C_q_, C-13), 120.8 (CH, C-12), 120.2 (C_q_, C-3), 114.0 (CH_2_, C-23); 66.9 x 2, 46.54 and 47.0 (4 x CH_2_, morpholine ring), 47.4 (C_q_, C-17); **DEPT**: 6 x CH_3_, 15 x CH_2_, 4 x CH (34 x C atoms); **MS** (m/z, %): 520.7 (19.1, M^+•^); **HR MS**: calcd. for C_34_H_52_N_2_O_2_: 520.4028, found: 520.4021.

**3-Oxo-3a-aza-A-homoolean-12-en-28-oic acid morpholide 12a** (afforded from acetone fraction): C_34_H_54_N_2_O_3_; mol mass: 538.81; yield: 329 mg (61.0%); m.p.: 277–280°C (ethanol); **IR** (KBr, ν, cm^-1^): 3450 (N-H), 1670 (C = O, lactam), 1630 (C = O, amide); ^**1**^ **H NMR** (CHCl_3_, δ, ppm): 5.69 (1H, s, N–H, lactam), 5.28 (1H, t, *J* = 3.4 Hz, C_12_-H), 3.70–3.54 (4 x 2H, m, morpholine ring), 1.27, 1.24, 1.14, 1.10, 0.93, 0.90, 0.78 (21 H, 7 x s, 7 x CH_3_); ^**13**^ **C NMR** (CHCl_3_, δ, ppm): 176.9 (C_q_, C-3, lactam); 174.9 (C_q_, C-28), 144.2 (C_q_,C-13), 121.4 (CH, C-12), 66.9 x 2, 46 4 and 47.0 (4 x CH_2_, morpholine ring), 47.4 (C_q_, C-17); **DEPT**: 7 x CH_3_, 14 x CH_2_, 4 x CH (34 x C atoms); **MS** (m/z, %): 538.9 (23.3, M^+•^); **HR MS**: calcd. for C_34_H_54_N_2_O_3_: 538.4132, found: 538.4134.

### 2. *In vitro* tests of penetration enhancing activity

#### 2.1. Preparation of isonicotinic acid hydrazide solution

Isonicotinic acid hydrazide (0.1 g) was dissolved in methanol (15 mL) in volumetric flask (of 25 mL). Then concentrated hydrochloric acid (0.12 mL) was added and the obtained solution was filled up with methanol to 25.0 mL.

#### 2.2. Preparation of ointments with progesterone

Progesterone (1.0 g) was dissolved in ethanol (25.0 mL) and water (38.0 mL). Then Carbopol 980 (1.0 g) was added and the resulting mixture was stirred up for 120 minutes at room temperature in order to hydrate the acrylic acid. The excess of this acid was neutralized with a 10.0% solution of trometamol and stirred for a while. The model ointment, without progesterone, was prepared according to the above method without the usage of progesterone.

#### 2.3. Preparation of ointments with penetration enhancer

329 or 161 mg of the tested enhancer was dissolved in 10 mL of ethanol. Then 1.5 g (1.9 mL) of such solution was mixed with 4.5 g of ointment with progesterone described in 2.2.

#### 2.4. Preparation of lipophilic memebranes

Dodecanol (1.0 g) and a 4% solution of collodium in diethyl ether (the same volume as 1.0 g of dodecanol) were mixed. Then 1.5 mL of the above mixture of collodium was inserted into the cylinders of 50 mm diameter and left for 24 hours at room temperature. Next, 5 mL of bidistilled water was added into each cylinder and after 30 minutes the membranes were taken out and dried.

#### 2.5. Penetration of progestrone tests

A set of 5 artificial, stabilized lipophilic membranes were arranged consecutively on a plexiglass plate and covered with a plate-template. The tested ointment (0.01 g) was applied on the surface of the first membrane as defined by the stencil cavity. The set of membranes was inserted in the Fürst apparatus [28] and put into a thermostate in 37°C for 60 minutes. Next the membranes were separated and each membrane was separately dissolved in methanol (3.0 mL). The isoniazide solution (0.5 mL) was added to each of the obtained methanolic solutions and the mixtures were left in darkness for 30 minutes. The absorption of UV light with a wavelength λ_max_ of 395 nm (the absorption maximum of the resulted complex) was measured against the reference sample, which was preparared as above but without progesterone.

The percentage of progesterone in each membrane was estimated on the basis of the equatation [28]:

$$C=\frac{A\cdot v}{a_{1cm}^{1\%}\cdot M\cdot m}\cdot 100\%$$

*C*—percentage of progesterone absorbed within the membrane

*A*—absorance of the tested sample

$a_{1cm}^{1\%}$ —specific absorbance of progesterone (410.9)

*v*—volume of the tested sample (3.5 mL)

*M*—concentration of progesterone in an ointment [%]

*m*—mass of ointment applied on the membrane [g]

All of the obtained compounds containing the amide function at C-17 position were examined *in vitro* in order to estimate their ability to penetration activity of a model therapeutic substance into a system of synthetic lipophilic membranes simulating the corneous skin layer. An analogous test was performed for the standard substance, Azone, for a comparison. The results are presented in **Table 1**.

**Table 1:** The average percentage of progesterone absorbed from oinment into membranes 1–3, 4–5 and 1–5.

	The percentage of progesterone absorbed in membranes (%)					
nr of promoter and its concentr.	Σ 1–3	Σ 4–5	Σ 1–5	relative increase of absorbed progesterone		
				Σ 1–3	Σ 4–5	Σ 1–5
**without promoter**	56.76 ± 0.70	13.02 ± 0.50	69.78 ± 0.96	-	-	-
**Azone** (0.5%)	74.83 ± 1.54	10.73 ± 1.61	85.56 ± 1.30	+ 31.83	- 17.59	+ 22.61
**4a** (0.5%)	59.90 ± 0.73	14.62 ± 1.37	74.52 ± 1.82	+ 5.53	+ 12.29	+ 6.79
**4a** (1.0%)	79.54 ± 1.68	18.12 ± 1.36	97.66 ± 1.54	+ 40.13	+ 39.17	+ 39.95
**5a** (0.5%)	56.97 ± 1.31	12.59 ± 1.88	69.56 ± 1.75	+ 0.37	- 3.30	- 0.31
**5a** (1.0%)	71.89 ± 1.56	14.42 ± 1.61	86.31 ± 2.13	+ 26.66	+ 10.75	+ 23.69
**9a** (1.0%)	65.04 ± 0.99	20.32 ± 1.55	85.36 ± 1.82	+ 14.59	+ 56.07	+ 22.33
**10a** (1.0%)	59.75 ± 1.13	18.30 ± 1.80	78.05 ± 1.47	+ 5.27	+ 40.55	+ 11.85
**12a** (1.0%)	68.85 ± 1.57	21.57 ± 1.80	90.42 ± 1.95	+ 21.30	+ 65.67	+ 29.58
**4b** (0.5%)	67.43 ± 1.38	21.16 ± 1.28	88.59 ± 1.85	+ 18.80	+ 61.75	+ 26.96
**4b** (1.0%)	70.37 ± 1.54	13.51 ± 1.52	83.88 ± 0.39	+ 23.98	+ 3.76	+ 20.21
**5b** (0.5%)	66.12 ± 1.59	20.73 ± 1.31	86.85 ± 2.27	+ 16.49	+ 59.22	+ 24.46
**5b** (1.0%)	66.67 ± 1.59	16.56 ± 1.62	83.23 ± 2.05	+ 17.46	+ 27.19	+ 19.27
**4c** (1.0%)	65.70 ± 1.27	10.58 ± 1.42	76.28 ± 1.52	+ 15.75	- 18.74	+ 9.31
**5c** (1.0%)	58.09 ± 1.65	21.44 ± 0.75	79.53 ± 2.29	+ 2.34	+ 64.67	+ 13.97
**4d** (0.5%)	58.49 ± 1.42	18.29 ± 0.61	76.78 ± 1.28	+ 3.05	+ 40.48	+ 10.03
**4d** (1.0%)	68.27 ± 0.82	19.56 ± 0.82	87.83 ± 0.91	+ 20.28	+ 50.23	+ 25.87
**8d** (1.0%)	62.67 ± 1.38	15.89 ± 1.22	78.56 ± 1.59	+ 10.41	+ 22.04	+ 12.58
**4e** (0.5%)	56.47 ± 1.22	19.39 ± 0.62	75.86 ± 0.99	– 0.51	+ 48.92	+ 8.71
**4e** (1.0%)	67.46 ± 0.57	13.50 ± 0.95	80.96 ± 1.00	+ 18.85	+ 3.69	+ 16.02
**4f** (0.5%)	66.28 ± 1.29	11.10 ± 1.66	77.38 ± 1.42	+ 16.77	- 14.75	+ 10.89
**4f** (1.0%)	70.01 ± 1.84	18.59 ± 1.37	88.60 ± 1.59	+ 23.34	+ 42.78	+ 26.97

## Conclusions

It was proven, on the basis of the conducted analyses of the dependence of the obtained compounds’ structure and their biological activity as transdermal delivery promoters, that replacing of the carboxylic group at C-17 position with the amide function led to a group of compounds of high activity. Almost all of the obtained compounds in both concentrations increased the total amount of the model substance in a set of artificial membranes simulating epidermis, particularly in the external part of this model (*stratum corneum*). In most cases the increased concentration of the triterpenic promoter caused better penetration of progesterone into these memebrane leyers. In general, alkalyne hydrolysis of the acetoxy function within the acetoxyamides led to compounds with lower activity as transdermal penetration promoters in comparison to the appropriate amides of acetyloleanolic acid. Introducing an additional 11-oxo function into the molecule of triterpenic amide did not have an influence on the total amount of progesterone that was absorbed into the set of five membranes.

Our results proved that the most advantageous amide group positively influencing the total amount of progesterone absorbed in the whole system of lypophilic membranes were the N-methylpiperazine, the morpholine and the N,N-diethylamine moieties. A markedly increased amount of absorbed progesterone in all of the membranes, higher than in the case of applying of Azone, was observed for amides: **4a**, **5a** (both with the morpholine ring), **4b**, **5b** (both with the N-methylpiperazine ring and the acetoxy or the hydroxy group at C-3 position), **4d** (with the N,N-diethyl function and the acetoxy function at C-3 position) and **4f** (having two nitrogen atoms within the amide function and the acetoxy group at C-3 position). Particularly active penetration promoters belonging to the group of oleanolic or acetylooleanolic acid amides turned out to be morpholides. Further activation of promoting activity of these compounds was performed by transforming of the C-3 hydroxy function, and the most active compound was seven-membered A-lactam.

Among the tested triterpenes, morpholide **4a** in a 1.0% concentration caused an over 40% increase in the progesterone amount in the external part of the human skin model (membranes 1–3). Two of the tested enhancers in a concentration of 0.5% (**4b** and **5b**) and four derivatives in a concentration of 1.0% (**9a**, **12a**, **5c** and **4d**) caused an over 50% increase in the amount of the absorbed progesterone in the deeper parts of the *stratum corneum* model (membranes 4 and 5). Replacement of the C-3 hydroxyl group with the carbonyl or the hydroxyimino function as well as transformation of the last group into a lactam system resulted in better penetration of progesterone into the deeper membranes, with the best results for the compound with lactam system **12a** (65.67% for membranes 4 and 5).
